# HaploSNPer: a web-based allele and SNP detection tool

**DOI:** 10.1186/1471-2156-9-23

**Published:** 2008-02-28

**Authors:** Jifeng Tang, Jack AM Leunissen, Roeland E Voorrips, C Gerard van der Linden, Ben Vosman

**Affiliations:** 1 Laboratory of Bioinformatics, Wageningen University, PO Box 8128, 6700 ET Wageningen, The Netherlands; 2 Plant Research International, PO Box 16, 6700 AA Wageningen, the Netherlands

## Abstract

**Background:**

Single nucleotide polymorphisms (SNPs) and small insertions or deletions (indels) are the most common type of polymorphisms and are frequently used for molecular marker development. Such markers have become very popular for all kinds of genetic analysis, including haplotype reconstruction. Haplotypes can be reconstructed for whole chromosomes but also for specific genes, based on the SNPs present. Haplotypes in the latter context represent the different alleles of a gene. The computational approach to SNP mining is becoming increasingly popular because of the continuously increasing number of sequences deposited in databases, which allows a more accurate identification of SNPs. Several software packages have been developed for SNP mining from databases. From these, QualitySNP is the only tool that combines SNP detection with the reconstruction of alleles, which results in a lower number of false positive SNPs and also works much faster than other programs. We have build a web-based SNP discovery and allele detection tool (HaploSNPer) based on QualitySNP.

**Results:**

HaploSNPer is a flexible web-based tool for detecting SNPs and alleles in user-specified input sequences from both diploid and polyploid species. It includes BLAST for finding homologous sequences in public EST databases, CAP3 or PHRAP for aligning them, and QualitySNP for discovering reliable allelic sequences and SNPs. All possible and reliable alleles are detected by a mathematical algorithm using potential SNP information. Reliable SNPs are then identified based on the reconstructed alleles and on sequence redundancy.

**Conclusion:**

Thorough testing of HaploSNPer (and the underlying QualitySNP algorithm) has shown that EST information alone is sufficient for the identification of alleles and that reliable SNPs can be found efficiently. Furthermore, HaploSNPer supplies a user friendly interface for visualization of SNP and alleles. HaploSNPer is available from .

## Background

Single nucleotide polymorphisms (SNPs) and small insertions or deletions (indels) are the most common type of genetic polymorphisms and well suited for molecular marker development due to their abundance within the genome and their slow mutation rate [1]. SNPs have become very popular for haplotype reconstruction [2]. The common approach to haplotype reconstruction is partitioning the genome into blocks that are in high linkage disequilibrium, based on the analysis of genome-wide SNPs, and structuring haplotypes in each block with limited diversity. This approach has been implemented in a number of programs, such as HAP[3].

Haplotypes can also be reconstructed for specific genes, based on the SNP present in the gene [4]. Haplotypes in this context represent the different alleles of a gene. It is possible to reconstruct alleles with SNPs that are identified in multiple EST sequences of specific genes: several closely linked SNPs from EST sequences of a gene can completely define alleles of the gene [5-7]. A set of SNPs discriminating all identified alleles can be used to study the association between candidate gene genotypes and phenotypes, and to select individuals with specific genotypes. The computational approach to SNP mining is increasingly fruitful because of the continuously increasing number of sequences deposited in databases. Several software packages have been developed to mine for SNPs [8-12]. However, sequences in public repositories often do not store their trace or quality files. Some tools [8,9,11,12] can not process publicly available sequences because they require sequences with trace or quality files, or even the corresponding genomic sequences. Only few software tools can detect SNPs from sequence information alone [5,10]. QualitySNP [5] is the only tool that combines SNP detection with the reconstruction of alleles from public EST data without the requirement for trace/quality files or genomic sequences. In this program, gene haplotypes representing alleles are defined by a mathematical algorithm and based on potential polymorphisms. Reliable SNP are identified using these constructed alleles and a confidence score that is calculated based on sequence redundancy in high and low quality regions. SNPServer [13] is the only web-based tool for SNP discovery that permits the real-time detection of SNPs related to any specified sequence of interest. This tool builds on the use of autoSNP [10] that utilizes the frequency of occurrence of a polymorphism and co-segregation of multiple SNPs to identify reliable SNPs. However, autoSNP cannot distinguish paralogs, which is a major cause for false detection of SNPs [5,14]. Moreover, compared to QualitySNP autoSNP detects many more false positive SNPs and requires more calculation time, in particular for large datasets [5].

In this paper we describe HaploSNPer, a web-based tool for the reliable detection of alleles and SNPs. It is based on finding homologous sequences in user-specified sequence databases using a user-supplied seed sequence, or on a collection of input sequences. HaploSNPer combines the QualitySNP algorithm with database search and sequence alignment tools into an efficient pipeline.

## Implementation

### The pipeline

**Figure 1:**
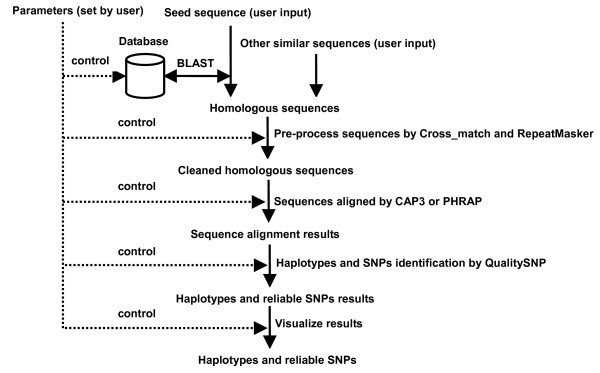
Flowchart of the HaploSNPer pipeline.

HaploSNPer includes BLASTN of the BLAST package [15] to find homologous sequences, CAP3 [16] or PHRAP [17] to align them, and QualitySNP to detect alleles and reliable SNP based on the alignment information (Figure 1). Extra programs, e.g. Cross_match [18] to remove vector sequences and RepeatMasker [19] for masking repeats are also included in HaploSNPer and can be activated upon request. HaploSNPer is implemented in PHP4 as a web-based service running on an Apache 2.0 server on a Linux system. The core application is a C-shell script that controls the performance of BLAST, CAP3 or PHRAP and QualitySNP. After a job has finished, the results are returned as HTML pages by a PHP script.

**Figure 2:**
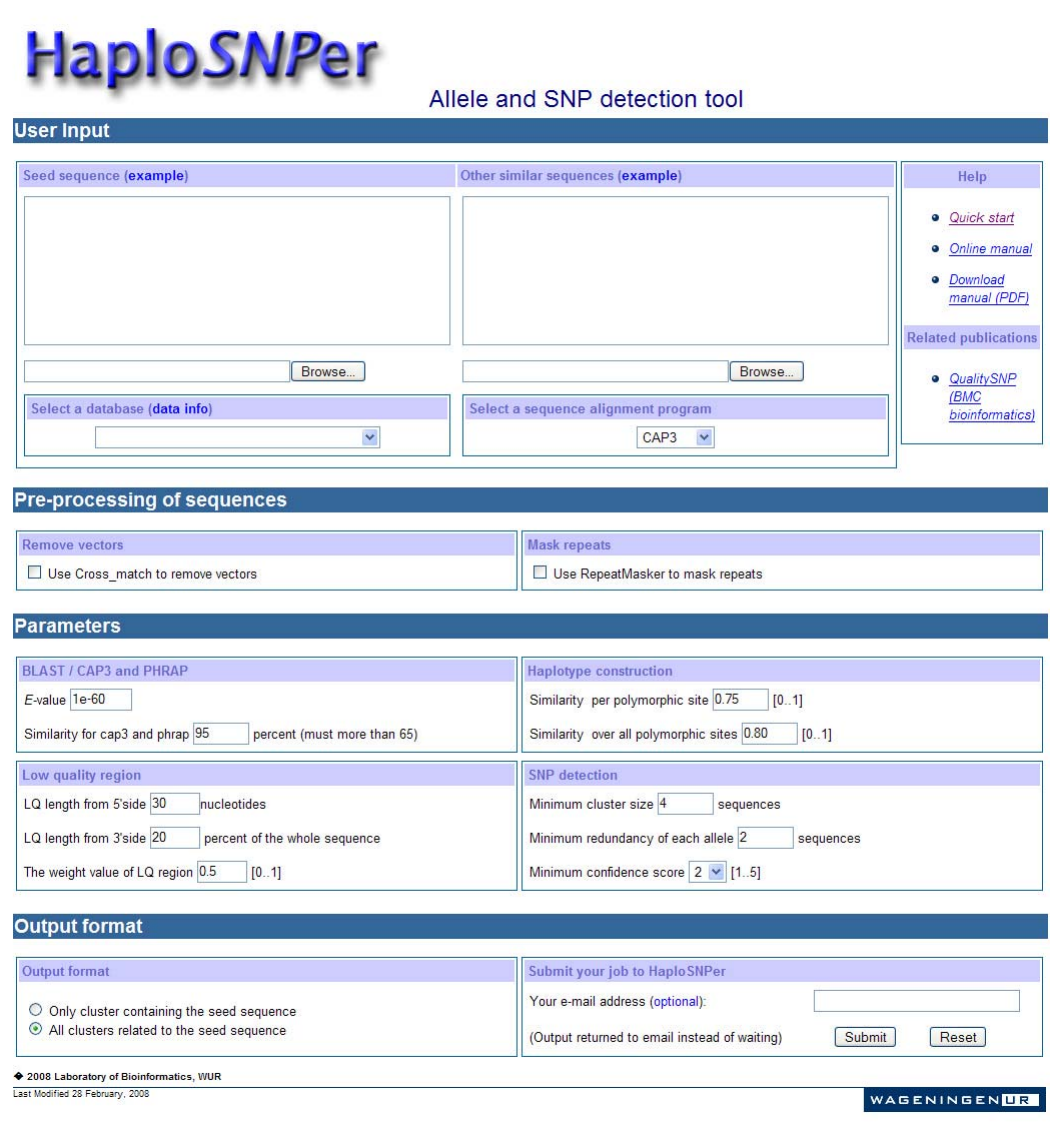
**Interface of HaploSNPer**. The meaning of all parameters is described in detail in [5] as well as in the on-line manual [23]. A set of example sequences are available at the HaploSNPer website at URL [23].

HaploSNPer is a user-friendly and flexible tool (Figure 2), which can run in either interactive or batch mode. In the latter case the results are returned by email. Also the input is flexible: users can submit either one sequence (in FASTA format) as the seed sequence, or a seed sequence in combination with a number of other homologous sequences. This last option is especially useful when users want to include homologous sequences that are not yet available in public databases. Additional sequences homologous to the seed sequence will be retrieved from the selected databases and all sequences will be used to identify alleles and SNPs related to the seed sequence. HaploSNPer can also process a dataset containing a number of homologous or similar sequences without the use of an additional database.

### Algorithms

#### 1. Haplotype reconstruction

In the QualitySNP algorithm, a potential haplotype is defined as a group of sequences within a cluster that have the same nucleotide at every polymorphic site [5]. For haplotype reconstruction, the similarity between a candidate sequence and a haplotype group at each single potential SNP is calculated and compared with a threshold to determine whether the nucleotide at that SNP position is identical in the candidate sequence and the haplotype group; then the similarity over all potential SNPs is compared with a second threshold to determine whether the candidate sequence can be reliably assigned to the haplotype group. By using the similarity per polymorphic site as well as the similarity over all polymorphic sites, alleles represented by the haplotype groups can even be reconstructed reliably with sequences containing sequencing errors by setting appropriate threshold values.

#### 2. Recognition of paralogs

Clusters containing paralogous sequences can be expected to contain more polymorphisms than clusters with only allelic sequences. A method based on the number and frequency of polymorphisms may therefore separate paralogs from alleles, such as implemented in POLYBAYES [14]. However, some EST clusters show a larger than average number of SNPs because some genes or regions of genes evolve more rapidly than others. These SNPs present allelic variations but will be mistaken for variations between paralogs by such an approach. The haplotypes that are initially identified by QualitySNP are potential haplotypes that may be groups of alleles and paralogs. In QualitySNP paralogs are distinguished from alleles based on the difference in SNP numbers between the potential haplotypes of the same cluster [5]. The standard deviation (D-value) of the number of potential SNPs among haplotypes in a cluster is calculated and used to assess the probability that the cluster contains haplotypes that are in fact not alleles but paralogs.

With increasing D-value the difference in number of SNPs among haplotypes is larger, so there is a higher probability of including alleles as well as paralogs in the cluster. At lower D-values, the probability that all the potential haplotypes found by QualitySNP are indeed alleles of a gene is higher, so most clusters with low D-values will contain few or no paralogs.

### Parameters

Several parameters can be set to tailor the performance and output of HaploSNPer to the specific requirements of the user (Figure 2). A database corresponding to the species of interest can be selected as the target database from the list provided by HaploSNPer. These databases contain all publicly available EST sequences extracted from the EMBL database and will be updated regularly. Currently HaploSNPer links to databases of nine animal and thirteen plant species. CAP3 or PHRAP can be chosen for sequence alignment. For SNP mining, CAP3 is recommended as it uses individual sequence overlap for cluster construction, while PHRAP tends to extend the consensus sequence by overlap. However, PHRAP is much faster than CAP3 [20]. BLASTN of the BLAST package that is a widely used tool for searching DNA databases for sequence similarities is used to search for sequences similar to the input (seed) sequence. The *E*-value of BLASTN is to find significantly similar sequences [14]. A series of thresholds of the *E*-value have been tested on several sequences; an *E*-value of 1e-60 usually results in the selection of sequences sufficiently similar to the seed sequence, and this value is set as default in HaploSNPer. As the *E*-value of BLASTN increases with the increasing size of both the query and the database, it can be adjusted by the user according to the outcome of trial runs using different *E *values and according to their experience.

Several parameters control the performance of the QualitySNP part of the pipeline [5]. The threshold for similarity per polymorphic site can be set based on the expected percentage of good quality sequences; a threshold of 75% achieved satisfactory results in our previous study [5], and also is the default value in HaploSNPer. The threshold for similarity over all polymorphic sites can be set according to the (assumed) similarity between the alleles. Setting these thresholds too low may result in several different alleles or even paralogous sequences being classified as a single haplotype, while too high settings will result in the separation of allelic sequences into different haplotypes because of sequencing errors.

The extent of the low-quality regions at the 5' and 3' ends of the sequences can be specified; these regions require more redundant information than high-quality regions. Based on examination of public EST sequences, we found that the 5' low-quality region is generally around 30 nucleotides in length, while the 3' low-quality region is about 20% of the sequence length [5].

HaploSNPer reports the D-value for every cluster, but does not use it as a selection criterion. As discussed in the previous section, higher D-values indicate a higher probability that a cluster contains paralogous as well as allelic sequences. In our previous study on a potato dataset [5], clusters with a D-value below 0.6 were shown to be generally composed of allelic sequences only. The choice of an appropriate threshold for the D-value requires additional study for each specific application [5]; however, the D-value can always be used to order clusters for probability of the presence of paralogous sequences.

## Results and discussion

The output produced by HaploSNPer consists of three parts. The first displays the settings of the parameters; the second part lists the information on clusters, haplotypes and the statistic information on SNPs, and the third part displays the haplotypes, SNP and sequence alignment for each cluster.

**Figure 3:**
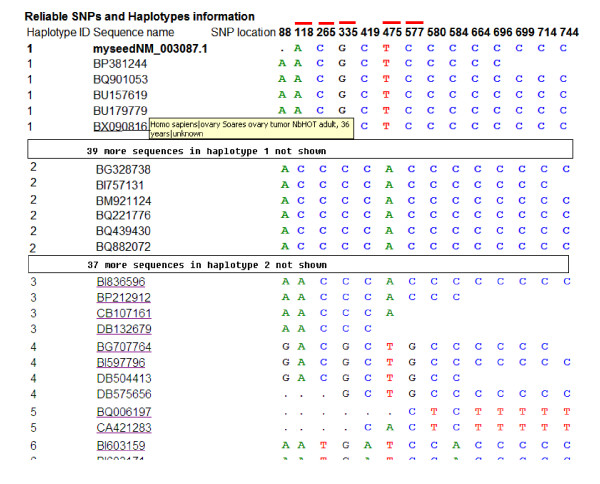
**Reconstruction of reliable haplotypes and prediction of reliable SNPs from the human gamma synuclein gene**. If additional information, such as cultivar/strain, tissue/clone and unigene ID of any sequence is available, it is shown when positioning the mouse over the sequence name, as illustrated for [EMBL: BX090816]. Five SNPs (in the consensus sequence 118,265,335,475 and 577) indicated with red rectangles are present in dbSNP (corresponding to rs1800373, rs11550192, rs760113, rs9864 and rs11550193 respectively).

To illustrate the performance of the program, we analyzed the human SNCG (gamma synuclein; breast cancer-specific protein 1) gene, a member of the synuclein family of proteins, which is believed to be involved in the pathogenesis of neurodegenerative diseases and associated with various cancers, in particular with breast cancer. The reference mRNA (NM_003087.1) of the SNCG gene obtained from the RefSeq database [21] is used as the seed sequence and the default settings of all parameters are used to control the performance of HaploSNPer. Sixty potential SNPs were identified with each allele represented by more than one sequence, of which 14 were considered as reliable SNP using the default criteria. Five of these SNPs at positions 118, 265, 335, 475 and 577 of the consensus sequence correspond to known SNP rs1800373, rs11550192, rs760113, rs9864 and rs11550193 present in dbSNP (version human_9606, April.19^th ^2007) respectively (Figure 3). The other 9 SNPs are newly identified polymorphisms, which may be evaluated in suitable materials. Six reliable alleles were identified. Allele reconstruction and SNP identification can be checked by clicking the link "show sequence alignment information". Reliable haplotypes and SNP are shown in Figure 3. The cluster is considered not to contain paralogs since the D-value of this cluster (0.5435) is relatively low. This was confirmed by a BLAST search: the consensus sequence of the cluster has only one high scoring hit on both the human genome and transcripts database of NCBI, indicating that the cluster represents a single locus and that the different haplotypes represent true alleles. Based on this result, several new SNPs can be selected to create allele-specific markers suitable for discriminating the alleles. The six alleles can be identified using the five validated SNP only (Figure 3).

**Figure 4:**
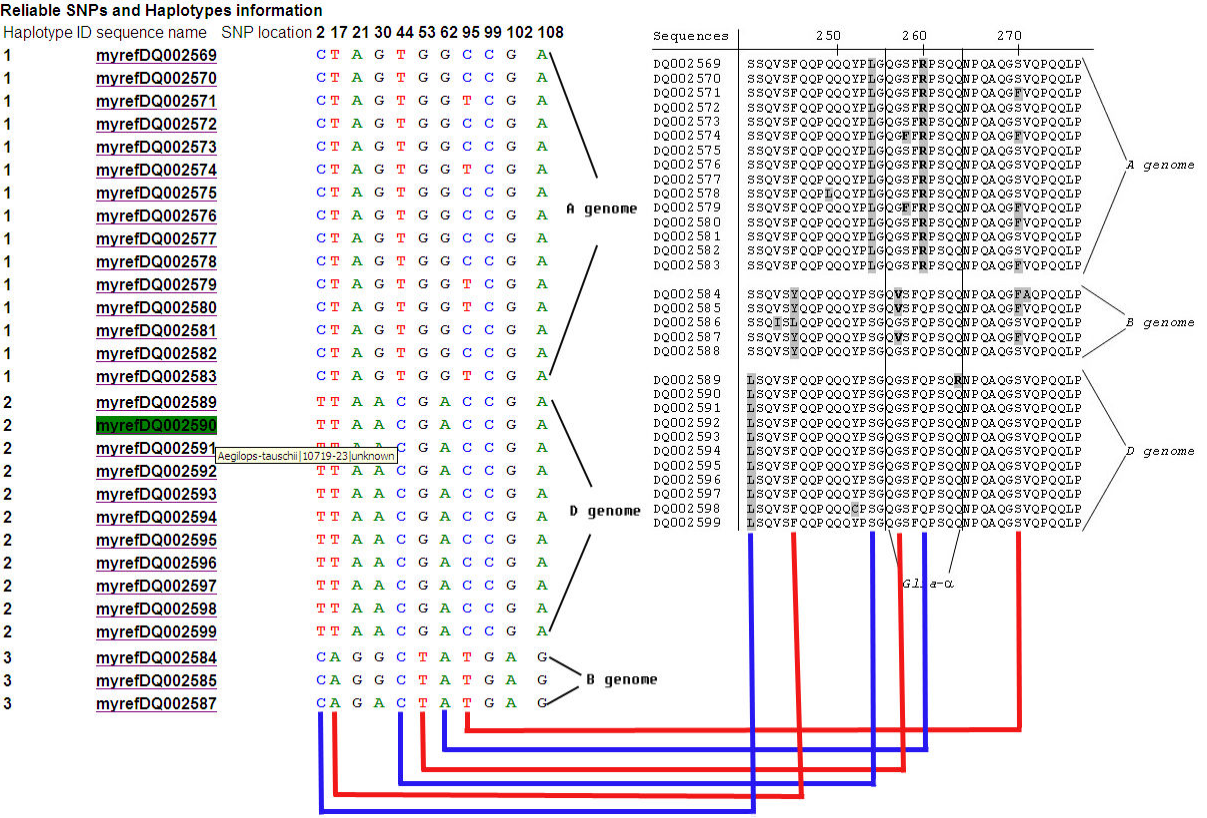
**Reconstruction of reliable haplotypes and prediction of reliable SNPs from the glia-a domain of alpha-gliadin genes of wheat**. The amino acid sequence alignment of the glia-a domain in the right part of the figure is from the figure 6 of [22]. Genome haplotypes reconstruction and reliable SNPs of the glia-a in the left part of the figure are from the output of HaploSNPer. Red and blue lines link the reliable non-synonymous SNPs predicted by HaploSNPer to amino acid sequence variations in [22].

As a second example of the potential of HaploSNPer we analyzed the wheat alpha-gliadin gene sequences described by [22], to show that HaploSNPer can discriminate haplotypes originating from different genomes in an allopolyploid species. The alpha-gliadin genes of wheat are a gene family associated with celiac disease [22], and are present on each of the three genomes (A, B and D) of hexaploid wheat. With HaploSNPer, the same A, B and D genome haplotype reconstruction (Figure 4) was obtained as reported in Figure 6 of [22] on the same sequences. Two sequences [GenBank: DQ002586, GenBank: DQ002588] reported by [22] for the B genome deviated so much that HaploSNPer classified them into two haplotypes consisting of only a single sequence, which were then filtered out. All six non-synonymous SNPs described [22] were detected by HaploSNPer. Five synonymous SNPs were detected as well (Figure 4). With this HaploSNPer output several SNPs could be selected as genome-specific markers to discriminate the A, B and D genome alpha-gliadin gene sequences.

## Conclusion

Through extensive testing we have shown that HaploSNPer (and the underlying QualitySNP algorithm) can efficiently detect reliable SNPs, reconstruct haplotypes and therefore identify different alleles using only EST sequence information. Furthermore, HaploSNPer supplies a user friendly interface for visualization of SNPs and alleles, which supplies the selection of informative SNP and allele-specific markers.

## Availability and requirements

Project name: HaploSNPer;

Project home page: ;

Operating system(s): platform independent;

Programming languages: C, PHP, C-shell

Any restrictions to use by non-academics: none

## Authors' contributions

BV, JL and JT identified the need to develop the program. JT designed the program and wrote the source code. All authors contributed to the overall design and feature requirements, and participated in the drafting of the manuscript and approved the final version.
